# A Simplified mAb-Based Antigen Detection Assay for Rapid Serotyping of Foot-and-Mouth Disease Virus

**DOI:** 10.3390/v17060761

**Published:** 2025-05-27

**Authors:** Mohammad A. Kashem, Thanuja Ambagala, Kate Hole, Ming Yang, Charles Nfon, Shawn Babiuk

**Affiliations:** National Centre for Foreign Animal Disease, Canadian Food Inspection Agency, Winnipeg, MB R3E 3M4, Canada; mohammad.kashem@inspection.gc.ca (M.A.K.); thanuja.ambagala@inspection.gc.ca (T.A.); kate.hole@inspection.gc.ca (K.H.); ming.yang@inspection.gc.ca (M.Y.); charles.nfon@inspection.gc.ca (C.N.)

**Keywords:** FMDV, DAS-ELISA, mAbs, specificity, sensitivity, cross-reactivity

## Abstract

Foot-and-mouth disease (FMD) is a devastating infectious viral disease of cloven-hoofed animals. Differentiating FMD from other vesicular diseases is difficult based on only clinical symptoms, requiring an appropriate laboratory diagnostic test. The double-antibody sandwich (DAS)-ELISA is a reliable diagnostic technique for antigen detection and serotyping of FMDV. However, classical DAS-ELISAs use polyclonal antibodies (pAbs), which are inconsistent in yields and limited in large-scale applications compared to hybridoma cell-secreted laboratory-made monoclonal antibodies (mAbs). Therefore, this study aimed to develop simplified and sensitive FMD serotype-specific DAS-ELISAs using HRP-conjugated mAbs and a TMB substrate. Six FMDV serotype-specific mAb-DAS-ELISAs were developed. All assays were optimized using BEI-inactivated FMD antigens. Real-time reverse-transcriptase PCR (RRT-PCR) was also used to verify the detection efficiency of all assays. Known negative and positive 10% tissue suspensions of different animal origins were examined to calculate the diagnostic specificity (DSp) and sensitivity (DSe). Serotype-specific mAb-DAS-ELISAs demonstrated 100%, 97%, 97%, 99%, 99%, and 94% DSp and 100%, 95%, 90%, 95%, 100%, and 100% DSe for serotypes O, A, Asia-1, SAT-1, SAT-2, and SAT-3, respectively. The detection efficiency of mAb-DAS-ELISAs was better than that of classical DAS-ELISAs. Also, all assays demonstrated minimal cross-reactivity and optimal reproducibility. Therefore, the mAb-DAS-ELISAs developed in this study could be useful for detecting and serotyping FMDV and ultimately replacing the classical DAS-ELISA.

## 1. Introduction

Foot-and-mouth disease (FMD), a highly infectious acute viral disease, affects cloven-hoofed animals [1,2,3,4]. The causal agent of FMD is the FMD virus (FMDV), which belongs to the family *Picornaviridae* under the genus *Aphthovirus* [5,6]. FMDV is antigenically highly variable and has seven distinct serotypes, namely O, A, C, Asia-1, Southern African Territories (SAT)-1, SAT-2, and SAT-3 [7,8,9,10]. The disease is endemic or sporadic in many countries globally, resulting in massive economic losses due to trade restrictions and the costs required to recover from outbreaks [11]. FMDV is distributed in seven geographic pools with an uneven distribution of serotypes and strains [9,12,13]. While FMD is geographically restricted [10,13], its highly contagious nature makes it feared as a transboundary disease and can cause serious economic consequences in countries currently free of the disease. Therefore, rapid detection and serotyping of FMDV is urgent to minimize global economic losses. Although suspected FMD cases can be identified by examining visible common clinical signs, confirmatory diagnosis without laboratory confirmation is highly [10,13] unreliable and difficult to differentiate from other vesicular diseases, including swine vesicular disease virus (SVDV), vesicular stomatitis virus (VSV), and Senecavirus A (SVA) [14,15,16,17,18,19].

Various laboratory techniques, including enzyme-linked immunosorbent assay (ELISA), virus isolation, virus neutralization test (VNT), complement fixation test (CFT), polymerase chain reaction (PCR), and sequencing, have been used to detect and identify FMDV serotypes [2,7,20,21,22,23,24]. Of these techniques, ELISAs are currently considered one of the most common methods for detecting and serotyping FMDV due to their greater efficiency, specificity, and sensitivity [25,26,27,28]. They are available in multiple formats, such as double-antibody sandwich (DAS), competition, and blocking ELISAs [20,29,30,31,32]. DAS-ELISAs assess test samples for the presence of viral antigens rapidly within a few hours without the need for virus isolation, which requires a longer time to isolate the virus [25]. To date, many modifications have been made for the performance improvement of classical DAS-ELISAs, particularly the development and application of monoclonal antibodies (mAbs) as trapping and detection antibodies [33].

In classical DAS-ELISAs, guinea pig polyclonal antibodies (pAbs) are used as detection antibodies [28,33]. Unfortunately, their use in DAS-ELISAs has some drawbacks, including the inconsistent yield of antibodies, batch-to-batch variation, and limited large-scale production [2,34]. On the contrary, specific hybridoma cell-secreted monoclonal antibodies (mAbs) serve as promising research and diagnostic reagents owing to their specificity, homogeneity, and large-scale production [2,35]. Thus, the primary purpose of this study was to develop a simplified and sensitive DAS-ELISA using hybridoma cell-secreted anti-FMDV mAbs. Anti-FMDV serotype-specific mAbs (HRP-conjugated) were used to develop DAS-ELISAs for rapid serotyping of FMDV.

## 2. Materials and Methods

### 2.1. Ethics Statement

The animal use and relevant experimental procedures in this study (animal use documents AUD C-02-007 SAT2 SAU 1/2000 2004-12-01, SAT3 ZIM 4/81 2004-08-16, ASIA SHAMIR 2003-04-22, AUD C-03-001 SAT3 ZIM 4/81 2003-12-08, AUD C-04-006 O UKG 11/2001 2006-11-02 AUD C-07-005 O serotypes 2007-11-02, A serotypes 2007-03-12, AUD C-07-008 O serotypes 2011-01-29, AUD C-08-009 O serotype 2008-10-28, AUD C-11-003 A IRN 1/2009 2012-07-28, Asia PAK 20/2003 2011-06-18, SAT1 ZAM9/08 2012-089-25, SAT2 EGY 6/2012 2012-11-24, SAT3 SAR 1/2006 2013-03-08, AUD C-14-002 A serotypes 2014-06-04, and AUD C-20-008 A serotype 2021-02-19) were approved by the Canadian Science Centre for Human and Animal Health Animal Care Committee (CSCHAH-ACC).

### 2.2. Samples

Tissue samples of different origins (pig, cattle, and sheep) were used in this study. Negative tissues (*n* = 75) were collected from healthy Canadian animals. For positive tissues, experimental infection of animals with different serotypes of FMDV (O UKG 11/2001 [*n* = 29]; A IRN 1/2009 and A VIT 15/2012 [*n* = 38]; Asia-1 PAK 20/2003 [*n* = 10]; SAT-1 ZAM9/08 and SAT-1 BOT1/68 [*n* = 20]; SAT-2 SAU 1/2000 and SAT-2 EGY 6/2012 [*n* = 26]; SAT-3 SAR 1/2006, and SAT-3 ZIM 4/81 [*n* = 33]) was performed, and tissues were collected on different days post-infection (dpi), mostly at 3–8 dpi. Tissues collected were cervical lymph nodes, mesenteric lymph nodes, tonsils, thymus, tongue, interdigit, heart, lungs, soft plate, hard plate, and hock joints.

### 2.3. Preparation of Tissue Suspensions

The tissue suspensions were prepared as previously described [36,37]. In brief, 0.5 g of tissue was homogenized using a Precellys 24 dual tissue homogenizer (2 × cycle 2: 5000 rpm, 3 × 10 s with 10 sec pause) (Bertin Technologies, Montigny-le-Bretonneux, France) along with 5 mL of Dulbecco’s phosphate-buffered saline (D-PBS) as a diluent to prepare 10% *w*/*v* suspensions. The suspension was clarified by low-speed centrifugation at 2000× g for 20 min at 4 °C, followed by harvesting the supernatant in small aliquots. These suspensions were treated as undiluted 10^0^ samples and stored at −70 °C for downstream analysis.

### 2.4. Production of FMDV Serotype-Specific mAbs

FMDV serotype-specific mAbs were produced in-house using hybridoma technology as described elsewhere [35,38,39]. Briefly, myeloma cells (P3X63 Ag8.653, ATCC, Rockville, MD, USA) were fused with splenocytes from female BALB/C mice immunized subcutaneously with binary ethyleneimine (BEI)-inactivated FMDV serotypes (20 µg/mouse), including O1/Campos, A22/Iraq, Asia-1/Shamir, SAT-1 Bot13/2015, SAT-2/SAU, and SAT-3 Zim 4/1981. Since FMDV serotype C is assumed to be extinct worldwide following the last detection in 2004 [40], we did not produce mAbs against serotype C. For each serotype, three fusions were conducted, and hybridomas were produced. Hybridoma cells were cultivated in BD Cell Quantum Yield medium (Gibco^TM^, Mississauga, ON, Canada; Cat# 220511) with 10% fetal bovine serum. The supernatants from hybridomas were screened using FMDV serotype-specific DAS-ELISAs. Using a limiting dilution method, all positive clones were subcloned in a 96-well culture plate and incubated at 37 °C with 5% CO_2_ for 14 days. Following subcloning, the mAbs were designated, and specific isotypes were characterized using a mouse monoclonal antibody isotyping kit (Roche, Mississauga, ON, Canada; cat# 11493027001). Hybridoma cell-secreted anti-FMDV mAbs were purified using a HiTrap Protein-G affinity column (GE Healthcare, CT, USA; cat#GE29-0485-81) based on the manufacturer’s instructions.

### 2.5. HRP Conjugation of FMDV mAbs

Mouse anti-FMDV serotype-specific mAbs were conjugated with horseradish peroxidase (HRP) using a Lighting-Link^®^ kit according to the protocol described earlier [26].

### 2.6. Development and Optimization of mAb-Based FMDV DAS-ELISA

A simplified mAb-based FMDV DAS-ELISA was developed as previously described, with slight modifications [36]. The optimal conditions for respective mAb-based FMDV DAS-ELISAs were determined by checkerboard titrations (Table 1). Briefly, a 96-well Maxisorp ELISA plate (Nunc-Immunoplate, Millipore Sigma, Toronto, ON, Canada) was coated with 100 µL/well of FMDV serotype-specific rabbit polyclonal antibodies (Table 1) diluted in 0.06 M carbonate buffer (pH 9.6), which act as capture antibodies. The plate was left overnight at 4 °C. Following 5^x^ washes, 100 µL/well of serotype-specific BEI-inactivated FMDV antigens diluted in 1^x^ casein blocking buffer (Sigma Aldrich, St. Louis, MO, USA; cat#B6429) or undiluted 10% stock tissue suspension (Table 1) was added to the plate and incubated. Unless otherwise stated, all incubations were performed at 37 °C for 1 h with gentle shaking, and all washes were carried out with 0.01 M phosphate-buffered saline (PBS) plus 0.05% Tween 20 (PBS-T), pH 7.2 ±0.1. After 5^x^ washes, 100 µL/well of HRP-conjugated FMDV serotype-specific mAbs (Table 1) diluted in 1^x^ casein blocking buffer were added and incubated. Following a final 5^x^ wash, 100 µL/well of TMB substrates (equal volume of substrate A and B; SeraCare, Milford, MA, USA; cat# 5120-0038 and 5120-0049) was added and incubated for 10 min at RT in the dark with gentle shaking. The reaction was immediately stopped by adding 100 µL/well of TMB stop solution (SeraCare, Milford, MA, USA; cat# 5150-0021), and the optical absorbance of each well was measured at 450 nm using a SpectraMax PLUS (Molecular Devices, San Jose, CA, USA).

### 2.7. Classical FMDV DAS-ELISAs

Classical DAS-ELISAs were conducted as previously described [7]. Like the mAb-DAS-ELISAs, a 96-well Maxisorp ELISA plate (Nunc-Immunoplate, Millipore Sigma, Toronto, ON, Canada) was coated with 100 µL/well of FMDV serotype-specific rabbit polyclonal antibodies as capture antibodies (Table 1) diluted in 0.06 M carbonate buffer (pH 9.6). After overnight incubation at 4 °C, the plate was washed 5^x^ and 100 µL/well of serotype-specific inactivated FMDV antigens diluted in DAS buffer (PBS-T containing 2% normal bovine serum [Gibco^TM^, New Zealand origin, Mississauga, ON, Canada; cat# 16-170-086], 2% rabbit serum [Gibco^TM^, Mississauga, ON, Canada; cat# 16-120-099], and 2% phenol red indicator) or undiluted 10% tissue suspensions were added to the plate and incubated for 1 h at 37 °C with gentle shaking. After 5^x^ washes with PBS-T, 100 µL/well of guinea pig anti-FMDV polyclonal antibodies raised against purified inactivated antigens of each FMDV serotype diluted in DAS buffer, was added and incubated for 30 min at 37 °C with gentle shaking. Following another 5^x^ washes, 100 µL/well of HRP-conjugated donkey anti-guinea pig immunoglobulins (1:5000 dilutions in DAS buffer; Jackson ImmunoResearch Laboratories, Inc., West Grove, PA, USA, cat# 706-035-148) was added and incubated for 30 min at 37 °C with gentle shaking. After a final 5^x^ wash, 100 µL/well of ortho-phenylenediamine dihydrochloride (OPD) (Sigma Aldrich, St. Louis, MO, USA, cat# P-9187) diluted in ultrapure water was added and incubated for 10 min at RT in the dark with gentle shaking. Immediately after 10 min, the reaction was stopped by adding 2.0 M sulfuric acid, and the plate was read using a SpectraMax PLUS (Molecular Devices, San Jose, CA, USA) with an optical absorbance set at 490 nm.

### 2.8. Determination of the Cut-Off Value of FMDV mAb-DAS-ELISAs

Approximately seventy-five negative tissue suspensions (including oral epithelia, tongue, coronary bands, etc.) from different animal species were examined using serotype-specific FMDV DAS-ELISAs. The OD450 values from each assay were recorded, and the cut-off was calculated using the relation $\bar{x}$ + 3SD, where $\bar{x}$ represents the mean OD450 value of ~75 negative samples, and 3SD indicates three standard deviations. An OD450 value of 0.1 was set as a cut-off for all FMDV DAS-ELISAs. When the test sample OD450 was below 0.1, it indicated a negative test; an OD450 value of ≥0.1 represented a positive test [39].

### 2.9. Diagnostic Specificity (DSp) and Diagnostic Sensitivity (DSe) of FMDV mAb-DAS-ELISAs

The DSp and DSe of the serotype-specific FMDV mAb-DAS-ELISAs were calculated by examining known negative 10% tissue suspensions and known positive 10% tissue suspensions of different animal origins, respectively.

### 2.10. Cross-Reactivity Analysis of FMDV mAb-DAS-ELISAs

All mAb-based FMDV DAS-ELISAs were evaluated for cross-reactivity using antigens of different FMDV serotypes or other vesicular disease viral antigens, including swine vesicular disease virus (SVDV) and vesicular stomatitis virus (VSV). HRP-conjugated anti-FMDV serotype-specific detection mAbs and positive control antigens were used for this analysis.

### 2.11. Repeatability of FMDV mAb-DAS-ELISAs

The repeatability of FMDV DAS-ELISAs was examined using serotype-specific BEI-inactivated positive control antigens. Antigens were tested in duplicate on the same microplate or across different microplates of multiple batches. The percent coefficient of variation (%CV) was utilized to identify the repeatability of intra- and inter-plate data. The %CV was estimated using the formula (SD/$\bar{x}$) × 100%, where SD represents standard deviations and $\bar{x}$ represents the mean. A %CV of ≤10% and ≤15% was set for intra- and inter-plate assays, respectively.

### 2.12. Limits of Detection of FMDV mAb-DAS-ELISAs

Known positive tissue suspensions (*n* = 4 from each serotype) were diluted 1:2 to 1:64 and tested with mAb-DAS-ELISAs. The OD450 value from each dilution was tabulated to identify the detection limit of each assay.

### 2.13. Viral RNA Extraction

Viral RNA was extracted from known positive tissue suspensions as described previously [41]. Briefly, 55 µL of 10% tissue suspension (diluted or undiluted) was used to extract viral RNA using a MagMax^TM^-96 viral RNA isolation kit (Applied Biosystems, Mississauga, ON, Canada; cat# AMB18365) and the KingFisher Apex DX automated extraction system (ThermoFisher Scientific, Mississauga, ON, Canada) according to the manufacturer’s instructions. An internal positive control RNA (VetMax^TM^ Xeno^TM^, ThermoFisher Scientific, Cat# A29765) was added to the sample as an extraction control. Following extraction, the RNA was immediately stored at −70 °C for subsequent use.

### 2.14. Real-Time Reverse-Transcriptase Polymerase Chain Reaction (RRT-PCR)

Viral RNA was amplified by real-time RT-PCR (RRT-PCR) using previously described primers/probes for FMDV [42]. Briefly, 4^x^ TaqMan^TM^ Fast Virus 1-Step Master Mix (Applied Biosystems, cat# 4444434) was used in an FMDV/XENO duplex reaction. One 25 µL reaction mix included 11.75 µL of RNase-Free Water, 6.25 µL of 4^x^ TaqMan^TM^ Fast Virus 1-Step Master Mix, 1 µL of 25^x^ FMDV primer/probe mix (0.5 µM of forward primer, 0.5 µM of reverse primer, 0.2 µM of FAM-labeled probe), 1 µL of VetMAX^TM^ Xeno^TM^ internal positive control VIC^TM^ Assay (ABI, cat# A29767), and 5 µL of RNA template. RRT-PCR was performed with the following amplification setup: 1^x^ cycle at 50 °C (5 min) followed by 95 °C (20s), 40^x^ cycles at 95 °C (15s), and 60 °C for 45s (data collection).

### 2.15. Data Analysis

Each sample was duplicated in a 96-well microplate, including a negative (diluent) control. Raw OD values from each replicate were tabulated in Microsoft Office Excel 2016, and final OD values were achieved by subtracting the diluent control’s OD from the test sample ODs. The final OD values were transferred and organized into GraphPad Prism Version 10.2.2 for conducting statistical analysis and graphical presentation.

## 3. Results

### 3.1. Evaluation of Optimal mAb-Based FMDV Serotype-Specific DAS-ELISAs

**Figure 1:**
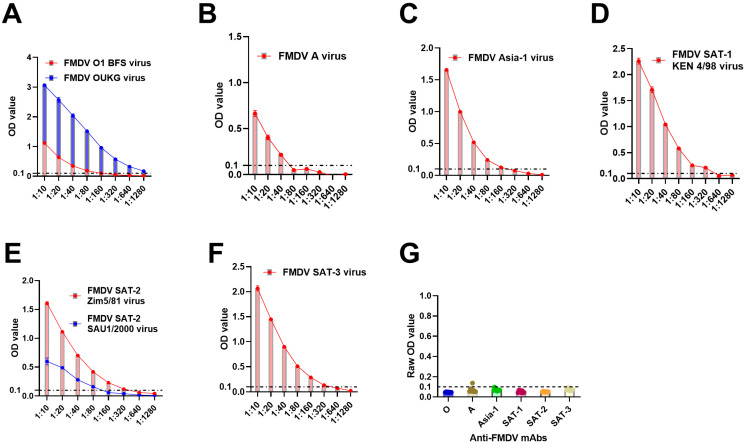
**FMDV was detected by HRP-conjugated serotype-specific anti-FMDV mAb-DAS-ELISAs.** (**A**) Diluted culture supernatants containing FMDV serotypes O (**A**), A (**B**), Asia-1 (**C**), SAT-1 (**D**), SAT-2 (**E**), and SAT-3 (**F**). (**G**) Baseline noise generated by HRP-conjugated mAbs. In (**A**–**F**), the *x*-axis shows FMD virus concentrations, and the *y*-axis indicates OD450 values. In (**G**), the *x*-axis represents the anti-FMDV mAbs used against assay buffer, and the *y*-axis shows raw OD450 values. The dashed line in all figures indicates the cut-off (0.1). Legends on the top of each figure represent FMDV strains.

To evaluate the detection efficiency of the optimal conditions for respective mAb-based DAS-ELISAs (Table 1), FMD viruses of the same serotype were diluted, ranging from 1:10 to 1:1280. The results demonstrated that serotype-specific DAS-ELISAs detected the FMDV serotypes with variable concentrations (Figure 1A–F). The data also showed a low baseline noise for all anti-FMDV mAbs used against only assay buffer (Figure 1G), suggesting that HRP-conjugated serotype-specific mAbs could be good for FMDV DAS-ELISAs.

### 3.2. DSp of mAb-Based FMDV DAS-ELISAs

**Figure 2:**
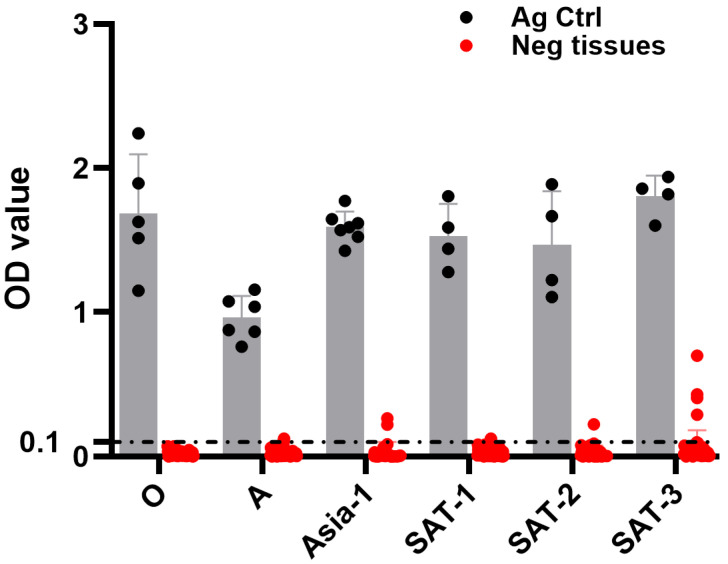
**DSp of FMDV serotype-specific mAb-DAS-ELISAs.** Known negative tissue suspensions (10%) prepared from porcine, bovine, and ovine tissues were examined by serotype-specific HRP-conjugated mAb-DAS-ELISAs. The same tissue suspensions were used for multiple serotypes, such as FMDV/O (*n* = 75), FMDV/A (*n* = 74), FMDV/Asia-1 (*n* = 64), FMDV/SAT-1 (*n* = 72), FMDV/SAT-2 (*n* = 71), and FMDV/SAT-3 (*n* = 67). The *x*-axis represents the FMDV serotypes, and the *y*-axis shows raw OD450 values. BEI-inactivated FMDV serotype-specific antigens were used as positive controls. The gray bars show BEI-inactivated antigen controls, and the red bars represent known negative tissue suspensions. The dashed line indicates the cut-off (0.1).

To evaluate the DSp of the HRP-conjugated serotype-specific anti-FMDV mAb-DAS-ELISAs, known negative tissue suspensions (*n* = 75, 74, 64, 72, 71, and 67 for serotypes O, A, Asia-1, SAT-1, SAT-2, and SAT-3, respectively) were tested. Known BEI-inactivated FMDV antigens for each serotype were used as positive controls. The study exhibited a 94–100% DSp. Particularly, 100% DSp was observed in the serotype O-specific mAb-DAS-ELISA (Table 2 and Figure 2). Serotype A-, Asia-1-, SAT-1-, and SAT-2-specific mAb-DAS-ELISAs demonstrated 97%, 97%, 99%, and 99% DSp, respectively (Table 2 and Figure 2). These data, therefore, suggested that FMDV serotype-specific DAS-ELISAs were specific to antigens of interest in test specimens.

### 3.3. DSe of mAb-Based FMDV DAS-ELISAs

**Figure 3:**
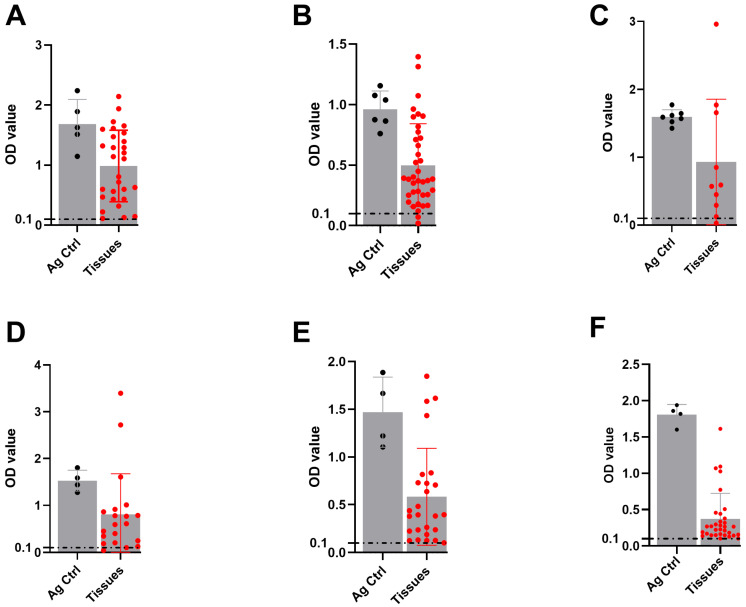
**DSe of FMDV serotype-specific mAb-DAS-ELISAs.** Known positive tissue suspensions (10%) prepared from experimentally infected animal tissues (porcine, bovine, and ovine) were examined by HRP-conjugated mAb-DAS-ELISAs specific for FMDV serotypes O (**A**), A (**B**), Asia-1 (**C**), SAT-1 (**D**), SAT-2 (**E**), and SAT-3 (**F**). Inactivated FMDV serotype-specific antigens were used as positive controls. Variable sample numbers (*n*) across different serotypes, such as O (*n* = 29), A (*n* = 38), Asia-1 (*n* = 10), SAT-1 (*n* = 20), SAT-2 (*n* = 26), and SAT-3 (*n* = 33), were tested. The gray bars with black dots represent antigen controls, and the gray bars with red dots indicate known positive tissue suspensions. The dashed line indicates the cut-off (0.1).

To evaluate the DSe of serotype-specific FMDV mAb-DAS-ELISAs, known experimentally infected animal tissue suspensions (10%) of different origins were tested. Data analysis revealed 90% to 100% DSe. Specifically, 100% sensitivity was observed for serotype O-, SAT-2-, and SAT-3-specific assays (Table 2 and Figure 3A,E,F). On the other hand, 95%, 90%, and 95% DSe were recorded for serotypes A, Asia-1, and SAT-1, respectively (Table 2 and Figure 3B–D).

### 3.4. Limits of Detection

**Figure 4:**
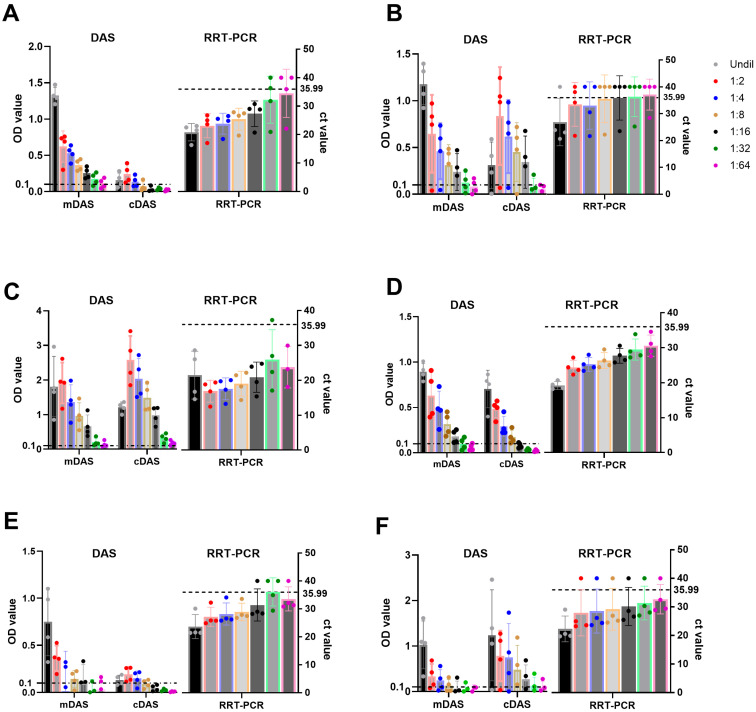
**Limits of detection of FMDV serotype-specific mAb-DAS-ELISAs, classical DAS-ELISAs, and RRT-PCR assays.** Known positive 10% tissue suspensions (*n* = 4), either undiluted or diluted (1:2 to 1:64), were examined by HRP-conjugated mAb-DAS-ELISAs, classical DAS-ELISAs, and RRT-PCR primer/probe assays specific to FMDV serotypes O (**A**), A (**B**), Asia-1 (**C**), SAT-1 (**D**), SAT-2 (**E**), and SAT-3 (**F**). The *x*-axis shows the type of assays, and the *y*-axis indicates OD450 values for mAb-DAS-ELISAs and classical DAS-ELISAs, and Ct values for RRT-PCR. The dashed line in all figures indicates the cut-off (0.1 OD450 for mDAS- and cDAS-ELISAs and 35.99 ct values for RRT-PCR). Legends in the top right corner indicate the sample dilution series (undiluted or diluted [1:2 to 1:64]). The color of dots in each bar represents the dilution series. mDAS: mAb-DAS-ELISA; cDAS: classical DAS-ELISA; RRT-PCR: real-time reverse-transcriptase PCR; Undil: undiluted. Each dot represents one individual sample.

To identify the limits of detection with an acceptable minimal sensitivity of mAb-DAS-ELISAs, a two-fold dilution (1:2 to 1:64) of strongly positive samples (*n* = 4) from each serotype was evaluated by mAb-DAS-ELISAs (mDASs) and classical DAS-ELISAs (cDASs). The results demonstrated that mAb-DAS-ELISAs detected antigens in 100% of undiluted tissue samples (4/4) for all serotypes (Table 3, Figure 4A–F). In contrast, classical DAS-ELISAs detected antigens in 100% undiluted tissues (4/4) for only serotypes Asia-1 and SAT1 (Table 3). In the case of diluted samples, serotype O-, Asia-1-, and SAT-1-specific mAb-DAS assays detected antigens up to 1:16 dilutions (Figure 4A,C,D), whereas serotype A, SAT-2, and SAT-3 assays showed less sensitivity once the sample was diluted (Figure 4B,E,F). At least one sample from serotypes A and SAT-3 went below the cut-off when diluted to 1:2. Similarly, less sensitivity for diluted samples was observed in classical DAS-ELISAs (Figure 4). However, the overall analytical sensitivity of mAb-DAS-ELISAs was better than that of classical DAS-ELISAs (Table 3). Thus, undiluted 10% tissue suspensions would be ideal for serotype-specific mAb-DAS-ELISAs.

In parallel, the accuracy of serotype-specific FMDV mDAS-ELISAs was evaluated by examining undiluted and diluted (1:2 to 1:64) known positive 10% tissue suspensions from each serotype (*n* = 4) using RRT-PCR. The Ct value cut-off was set at 35.99. A Ct value above 35.99 was treated as negative or suspicious. In this study, RRT-PCR data showed a similar trend in FMDV detection compared to mAb-DAS-ELISA results (Figure 4). RRT-PCR assay showed Ct values below 35.99 for all samples in 1:2 to 1:16 dilutions for serotypes O, Asia-1, and SAT-1 (Figure 4A,C,D), while for serotypes A, SAT-2, and SAT-3 (Figure 4B,E,F), the Ct values were a bit higher. Thus, the detection efficiency of mAb-DAS-ELISAs was comparable to RRT-PCR data and would be useful for rapid serotyping of FMDV.

### 3.5. Cross-Reactivity Analysis

**Figure 5:**
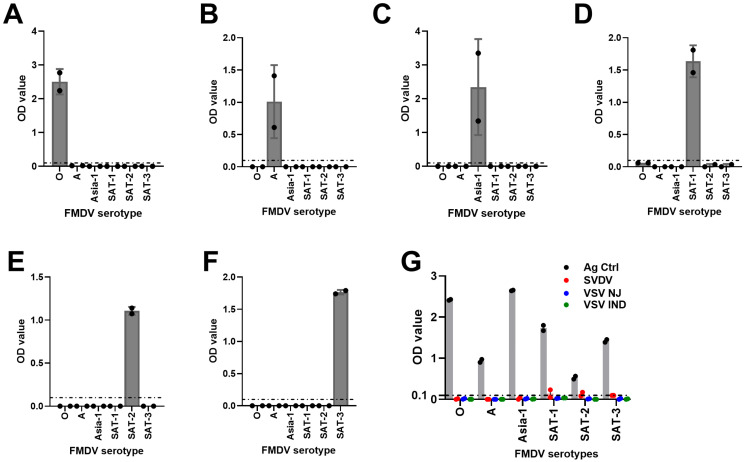
**Cross-reactivity analysis of FMDV serotype-specific mAb-DAS-ELISAs.** BEI-inactivated FMDV serotype-specific antigens of O (**A**), A (**B**), Asia-1 (**C**), SAT-1 (**D**), SAT-2 (**E**), and SAT-3 (**F**) were examined against each other. (**G**) FMDV serotype-specific mAb-DAS-ELISAs were also examined against antigens of other vesicular disease viruses (SVDV, VSV NJ, and VSV IND). Strong OD450 values in each figure indicate the antigen directed against the same serotype. The *x*-axis represents the FMDV serotypes, and the *y*-axis shows raw OD450 values. In (**G**), gray bars indicate antigen controls. The dashed line in all figures indicates the cut-off (0.1).

To assess whether HRP-conjugated mAbs cross-react with antigens of other FMDV serotypes or antigens of different viruses, a cross-reactivity analysis was carried out using known BEI-inactivated FMDV-positive antigens and other known vesicular disease viral antigens (SVDV, VSV NJ [New Jersey], and VSV IND [Indiana]). Data analysis revealed that mAb-based FMDV DAS-ELISAs were serotype-specific (Figure 5A–F) and also did not cross-react with other vesicular disease viral antigens except SVDV antigens for SAT-1- and SAT-2-specific assays (Figure 5G). Thus, these data demonstrated that the mAb-DAS-ELISAs developed in this study were FMDV serotype-specific.

### 3.6. Reproducibility of mAb-Based Anti-FMDV DAS-ELISAs

To examine the reproducibility of mAb-based anti-FMDV DAS-ELISAs, serotype-specific FMDV positive control antigens were tested in duplicates on the same microplate or across different microplates of different batches. The data were analyzed and presented as percentage coefficients of variation (%CV). A %CV of ≤10% and ≤15% was set for intra- and inter-plate assays, respectively. Our assays demonstrated 1.80 to 4.84%CV for intra-plate assays and 2.66 to 14.57%CV for inter-plate assays (Table 4). Thus, these data supported that mAb-based FMDV DAS-ELISAs were reproducible.

## 4. Discussion

Laboratory diagnostics are required to confirm a suspected FMD case from other vesicular diseases [2]. Although different laboratory techniques can be applied to identify FMDV from tissue suspensions, culture supernatants, or vesicular fluids, DAS-ELISA is the preferred approach for detecting and serotyping FMDV [28,43]. However, classical DAS-ELISAs are laborious and employ anti-FMDV pAbs as capture and detection antibodies [30]. It also requires an additional incubation step with either goat anti-mouse IgG-HRP conjugates or streptavidin-HRP conjugates when unconjugated or biotinylated detection antibodies are used [30]. In contrast, HRP can be directly conjugated to mAbs to detect FMDV in DAS-ELISAs, reducing the assay’s complexity. mAb-based DAS-ELISAs are found to be more specific, sensitive, and reproducible than classical DAS-ELISAs [33]. Therefore, this study aimed to develop simplified and sensitive FMDV DAS-ELISAs using hybridoma cell-secreted mAbs. The use of mAbs in DAS-ELISAs can allow for the removal of at least one incubation step, simplifying the assay format without compromising optimal sensitivity for rapid serotyping of FMDV.

Serotype-specific FMDV DAS-ELISAs were optimized to attain optimal assay conditions using HRP-conjugated FMDV serotype-specific mAbs. HRP-conjugated mAbs exhibited optimal assay sensitivity with a negligible baseline noise for all serotypes (Table 1). It was found that 1:15,000 (serotypes O and Asia-1), 1:1000 (serotypes A and SAT-3), and 1:7500 (serotypes SAT-1 and SAT-2) dilutions of HRP-conjugated serotype-specific mAbs were optimal for FMDV DAS-ELISAs. These assays also reported an optimal antigen load of 1:100, 1:5, 1:40, 1:10, 1:4, and 1:5 for serotypes O, A, Asia-1, SAT-1, SAT-2, and SAT-3, respectively. Most importantly, HRP-conjugation of anti-FMDV serotype-specific mAbs eliminated the use of polyclonal detection antibodies, which are inconsistent, as well as removed one extra incubation step, making the HRP-mAb-based approach simpler, more consistent, and more sensitive for rapid serotyping of FMDV.

The DSp of FMDV serotype-specific mAb-DAS-ELISAs was found to be between 94% and 100%. The overall DSp of mAb-based FMDV serotype-specific DAS-ELISAs developed in this study corroborates the earlier reports [33,34,44]. Nevertheless, these earlier reports did not examine DSp for all six serotypes. We also found that the DSp of our assays was higher than that of reports published by Ferris et al. [45,46].

The DSe of FMDV mAb-DAS-ELISAs was recorded between 90.00% and 100%. The overall DSe was found to be higher in our assays than the previously reported mAb-based DAS-ELISA [33,34,45,46]. The lowest DSe was observed for serotype Asia-1 (90%), which was due to the limitations of the samples. We only examined 10 known positive 10% tissue suspensions for Asia-1 (*n* = 10), which limits the overall sensitivity of the assay. Unfortunately, no further tissues for Asia-1 were available during the study to increase assay sensitivity. However, we are confident that including more known positive tissue suspensions would improve the overall DSe of Asia-1-specific mAb-DAS-ELISA.

The limits of detection of the mAb-DAS-ELISAs found in this study were comparable to RRT-PCR but higher than that of classical DAS-ELISAs. Serotype-specific mAb-DAS-ELISAs showed 100% detection efficiency in undiluted 10% known positive tissue suspensions for all serotypes tested. In contrast, classical DAS-ELISAs demonstrated 100% detection efficiency only in Asia-1- and SAT-1-specific assays. Only 75% detection was found for serotype O-, A-, and SAT-3-specific classical DAS-ELISAs, and the lowest detection efficiency was observed in the SAT-2-specific classical DAS-ELISA, which was 50%.

We also used a combination of two mAbs as detection antibodies, particularly for serotypes O and Asia-1, to maximize the detection coverage for multiple strains of the same serotype. The use of more than one mAb has merits because it is less likely that a field isolate will fail to have at least one epitope required to be recognized by the mAbs [2]. Our study revealed that using more than one mAb reduced the requirement of the final concentration of detection antibodies, as evidenced by higher antibody dilutions (1:15,000 for serotypes O and Asia-1). Unfortunately, we could not find a good combination of more than one mAb for other serotypes (A and SAT-1, -2, and -3) in this study.

All six serotype-specific DAS-ELISAs demonstrated non-reactivity to antigens of different FMDV serotypes or other vesicular disease viruses. Additionally, all mAb-DAS-ELISAs developed in this study were reproducible, which was evidenced by the %CV within the set limit of ≤10% for intra-plate assays and ≤15% for inter-plate assays.

## 5. Conclusions

In summary, six serotype-specific mAb-based DAS-ELISAs were developed for rapid serotyping of FMDV. All six assays demonstrated 94–100% DSp and 90–100% DSe with limited cross-reactivity. These assays also showed acceptable reproducibility. In addition, the mAb-DAS-ELISAs exhibited higher overall performance characteristics than the classical DAS-ELISA. Therefore, HRP-conjugated mAb-based DAS-ELISAs would be an ideal choice to replace the existing classical DAS-ELISA, which uses pAbs for rapid serotyping of FMDV. In future studies, the use of integrin alpha(v)beta6 will be assessed to replace the rabbit polyclonal FMDV capture antibodies in the serotype-specific mAb-DAS-ELISAs.

## Figures and Tables

**Table 1:** Reagents used to develop FMDV serotype-specific mAb-DAS-ELISAs.

	Reagents		Dilutions	Buffer	Serotype
1st Coat	Coating/capture antibodies				
	1.	Rabbit anti-FMDV O1 BFS 1860	1:10,000	CO3 (pH 9.6)	FMDV O
	2.	Rabbit anti-FMDV polyA (A24 Cruzeiro, A22 IRQ 24/64, A COL/85 and normal rabbit serum)	1:2500		FMDV A
	3.	Rabbit anti-FMDV Asia-1 Shamir	1:8000		FMDV Asia-1
	4.	Rabbit anti-FMDV SAT-1 Ken 4/98	1:4000		FMDV SAT-1
	5.	Rabbit anti-FMDV SAT-2 Zim 10/91	1:7000		FMDV SAT-2
	6.	Rabbit anti-FMDV SAT-3 Zim 4/81	1:8000		FMDV SAT-2
2nd Coat	Positive control antigens or test samples				
	1.	FMDV O1 BFS 1860	1:100	1^x^ Casein Sigma blocking	FMDV O
	2.	FMDV A24 Cruzeiro	1:5		FMDV A
	3.	FMDV Asia-1 Shamir	1:40		FMDV Asia-1
	4.	FMDV SAT-1 Ken 4/98	1:10		FMDV SAT-1
	5.	FMDV SAT-2 Zim 10/91	1:4		FMDV SAT-2
	6.	FMDV SAT-3 Zim 4/81	1:5		FMDV SAT-3
	10% stock tissue suspensions		Undiluted	-	Unknown (test sample)
Conjugates	Detection mAbs—HRP-conjugated				
	1.	F114S0-2-5-HRP	1:15,000	1^x^ Casein Sigma blocking	FMDV O
		F18-3-2-10-HRP	1:15,000		
	2.	F66 A22-14-HRP	1:1000		FMDV A
	3.	F36 ASIA 1 14-2-1-HRP	1:15,000		FMDV Asia-1
		F34 ASIA 1 8-2-1-HRP	1:15,000		
	4.	F78 SAT-1-12-2-1-HRP	1:7500		FMDV SAT-1
	5.	F76 SAT-2-10-2-1-HRP	1:7500		FMDV SAT-2
	6.	F80 SAT-3-41-HRP	1:1000		FMDV SAT-3
Substrate	TMB (solutions A and B) TMB stop solution		1:1 -	- -	- -

**Table 2:** Diagnostic specificity and sensitivity of mAb-DAS-ELISAs.

FMDV Serotype	Diagnostic Specificity				Diagnostic Sensitivity			
	*n*	(+)	(−)	%DSp	*n*	(+)	(−)	%Dse
**O**	75	0	75	**100**	29	29	0	**100**
**A**	74	2	72	**97**	38	36	2	**95**
**Asia-1**	64	2	62	**97**	10	9	1	**90**
**SAT-1**	72	1	71	**99**	20	19	1	**95**
**SAT-2**	71	1	70	**99**	26	26	0	**100**
**SAT-3**	67	4	63	**94**	33	33	0	**100**

**Table 3:** Analytical sensitivity of FMDV serotype-specific mAb-DAS-ELISAs versus classical DAS-ELISAs.

FMDV Serotype	mAb-DAS-ELISA (*n* = 4, Undiluted *)	Classical DAS-ELISA (*n* = 4, Undiluted *)
	Detection Efficiency	Detection Efficiency
**O**	100% (4/4)	75% (3/4)
**A**	100% (4/4)	75% (3/4)
**Asia-1**	100% (4/4)	100% (4/4)
**SAT-1**	100% (4/4)	100% (4/4)
**SAT-2**	100% (4/4)	50% (2/4)
**SAT-3**	100% (4/4)	75% (3/4)

* 10% known positive tissue suspensions.

**Table 4:** Reproducibility of FMDV serotype-specific mAb-DAS-ELISAs.

FMDV Serotypes	Intra-Plate Assay (*n* = 3)			Inter-Plate Assays (*n* = 3)		
	Stdv	Mean	%CV	Stdv	Mean	%CV
**O**	0.05	1.66	**3.31**	0.25	1.92	**13.11**
**A**	0.03	1.00	**3.36**	0.13	0.90	**14.57**
**Asia-1**	0.02	1.38	**1.80**	0.05	1.59	**3.15**
**SAT-1**	0.04	1.29	**3.48**	0.15	1.61	**9.33**
**SAT-2**	0.06	1.23	**4.84**	0.05	1.18	**4.30**
**SAT-3**	0.08	1.88	**4.05**	0.05	1.87	**2.66**

## Data Availability

All data supporting the findings of this study are available from the corresponding author upon reasonable request.
